# Is symptom-based diagnosis of lung cancer possible? A systematic review and meta-analysis of symptomatic lung cancer prior to diagnosis for comparison with real-time data from routine general practice

**DOI:** 10.1371/journal.pone.0207686

**Published:** 2018-11-21

**Authors:** Grace N. Okoli, Olga Kostopoulou, Brendan C. Delaney

**Affiliations:** 1 Clinical Lecturer in Primary Care, School of Population Health & Environmental Sciences, Faculty of Life Sciences and Medicine, King’s College London, London, United Kingdom; 2 Reader in Medical Decision Making, Department of Surgery and Cancer, Imperial College London, Norfolk Place, London, United Kingdom; 3 Chair in Medical Informatics and Decision Making, Imperial College London, Department of Surgery and Cancer, St Mary's Campus, London, United Kingdom; UT MD Anderson Cancer Center, UNITED STATES

## Abstract

**Background:**

Lung cancer is a good example of the potential benefit of symptom-based diagnosis, as it is the commonest cancer worldwide, with the highest mortality from late diagnosis and poor symptom recognition. The diagnosis and risk assessment tools currently available have been shown to require further validation. In this study, we determine the symptoms associated with lung cancer prior to diagnosis and demonstrate that by separating prior risk based on factors such as smoking history and age, from presenting symptoms and combining them at the individual patient level, we can make greater use of this knowledge to create a practical framework for the symptomatic diagnosis of individual patients presenting in primary care.

**Aim:**

To provide an evidence-based analysis of symptoms observed in lung cancer patients prior to diagnosis.

**Design and setting:**

Systematic review and meta-analysis of primary and secondary care data.

**Method:**

Seven databases were searched (MEDLINE, Embase, Cumulative Index to Nursing and Allied Health Literature, Health Management Information Consortium, Web of Science, British Nursing Index and Cochrane Library). Thirteen studies were selected based on predetermined eligibility and quality criteria for diagnostic assessment to establish the value of symptom-based diagnosis using diagnosistic odds ratio (DOR) and summary receiver operating characteristic (SROC) curve. In addition, routinely collated real-time data from primary care electronic health records (EHR), TransHis, was analysed to compare with our findings.

**Results:**

Haemoptysis was found to have the greatest diagnostic value for lung cancer, diagnostic odds ratio (DOR) 6.39 (3.32–12.28), followed by dyspnoea 2.73 (1.54–4.85) then cough 2.64 (1.24–5.64) and lastly chest pain 2.02 (0.88–4.60). The use of symptom-based diagnosis to accurately diagnose lung cancer cases from non-cases was determined using the summary receiver operating characteristic (SROC) curve, the area under the curve (AUC) was consistently above 0.6 for each of the symptoms described, indicating reasonable discriminatory power. The positive predictive value (PPV) of diagnostic symptoms depends on an individual’s prior risk of lung cancer, as well as their presenting symptom pattern. For at risk individuals we calculated prior risk using validated epidemiological models for risk factors such as age and smoking history, then combined with the calculated likelihood ratios for each symptom to establish posterior risk or positive predictive value (PPV).

**Conclusion:**

Our findings show that there is diagnostic value in the clinical symptoms associated with lung cancer and the potential benefit of characterising these symptoms using routine data studies to identify high-risk patients.

## Introduction

Lung cancer has the highest mortality rate of any cancer worldwide and constitutes more than 40% of all new cancer diagnoses [1]. Although survival rates in England have improved in the last 40 years, they remain lower than in comparable European countries. Improving early diagnosis is a key component of relieving the cancer burden [2]. It has been estimated that earlier diagnosis of the four commonest cancers in England (lung, breast, prostate and colorectal), would benefit over 11,000 patients each year [3]. The National Institute for Health and Care Excellence (NICE) 2015 urgent referral guidelines for suspected cancer, set the positive predictive value (PPV) threshold of clinical presentations for cancer at 3% [4]. In this study, we aim to determine the validity of symptom-based lung cancer diagnosis, using published studies, routine data from electronic health records and published prior risk models. A recent review of lung cancer diagnosis using ‘Risk Assessment Tools’ (RATs) found that there was insufficient validation, and that the inclusion of ‘epidemiological risk factors’ in the models, along with symptoms, created confounders [5]. In this review, we specifically assess symptoms associated with lung cancer diagnosis without epidemiological factors, to avoid confounding. We can then determine the prior risk using epidemiological models and calculate the posterior probability, or PPV, using Bayes’ theorem.

## Methods

### Systematic literature search

**Fig 1:**
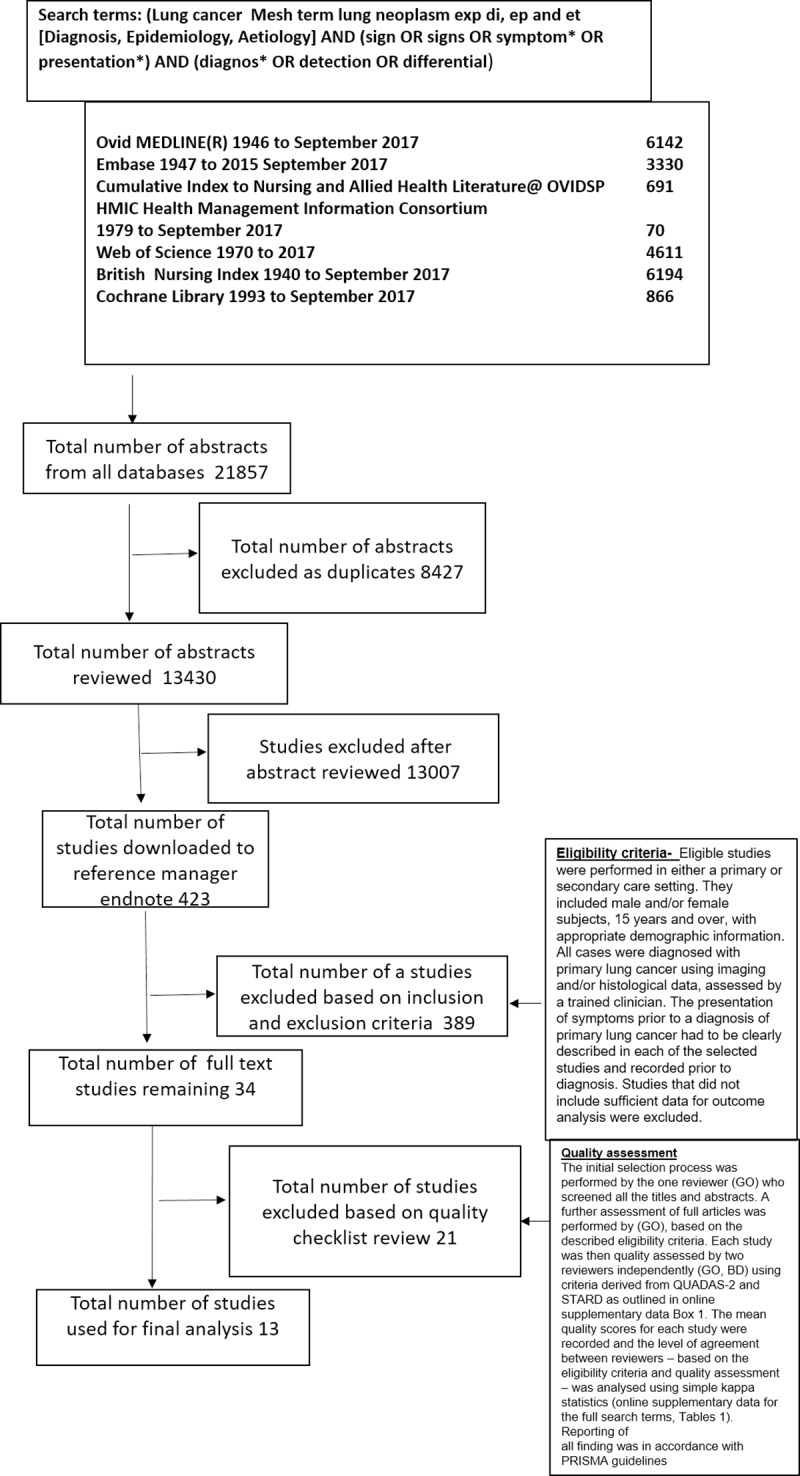
Prisma flow chart of database search.

We performed a systematic review and meta-analysis of studies reporting the sensitivity, specificity, predictive values, odds ratios or likelihood ratios for lung cancer in patients consulting their GP with symptoms prior to diagnosis. Searches were performed on 24^th^ September 2017 of seven databases using search terms specific for lung cancer diagnosis (Fig 1) presented using the prisma flow chart [6]. For prisma checklist and full search terms and outcomes, see S1 and S3 Tables.

### Eligibility criteria

Eligible studies were performed in either a primary or secondary care setting. They included male and/or female subjects, 15 years and over, with appropriate demographic information. All cases were diagnosed with primary lung cancer using imaging and/or histological data, assessed by a trained clinician. The presentation of symptoms prior to a diagnosis of primary lung cancer had to be clearly described in each of the selected studies and recorded prior to diagnosis. Studies that did not include sufficient data for outcome analysis using a 2x2 contigency table were excluded from the meta-analysis.

### Data collection from the TransHis primary care electronic health record

The Transition Project “TransHis” is an electronic patient record used by 230 general practices worldwide to collate data in real-time [7]. All patients whose initial consultations were subsequently linked to a diagnosis of lung cancer were assessed [8]. These allowed us to monitor the evolution of an initial presenting symptom to its final diagnosis [9]. Data extraction was performed on 24^th^ September 2017.

### Outcome analysis and statistical methods

For diagnostic analysis, we constructed 2x2 contingency tables for each study, using data collated prior to diagnosis [10]. For the meta-analysis, a random effects model for diagnostic accuracy was used to pool the data, as this accounts for differences in index test threshold, based on patient and/or clinical interpretation of presentations. A measure of the discriminatory power of the index test was calculated using diagnostic odds ratios (DOR). Heterogeneity in results across a study was assessed for each presenting symptom as a subgroup using Cochran's Q (Q*) and I-squared (I^2^) statistics [11, 12]. Summary Receiver Operating Characteristic (SROC) curves for each presenting symptom were plotted from pooled sensitivity against (1-pooled specificity) using Moses’ Model (weighted regression, inverse variance). The area under the curve (AUC) was used to measure diagnostic accuracy. STATA version 13 (STATACorp, USA) was used for the statistical analyses.

## Results

The search strategy shown in Fig 1, produced 13,430 unique references. A further review of these titles followed by abstracts and selection of those studies that met the inclusion criteria, resulted in the selection of 34 studies by the first reviewer (GO). A full text review of the 34 studies was performed by the first and second reviewer independently (GO and BD) with good agreement, kappa of 0.85 (0.430–0.938). After discussion, a final thirteen studies were selected. All findings were reported in accordance with PRISMA guidelines.

### Study strengths, limitations and bias assessment

The design and protocol used in each of the selected studies were subject to different types of bias (Table 1). The selected studies include six case series, three case-control and four cohort studies, summarised in Tables 2 and 3. Likelihood ratios (LR) are the most clinically useful outcome measures, as the LR is the probability of a cancer patient having the symptom divided by the probability of a non-cancer patient having that symptom. Table 3 details those studies where likelihood ratios could be calculated. Five of the selected studies included sufficient data to assess the diagnostic accuracy of symptoms associated with lung cancer using a dichotomous test approach. This data was compared with LRs from TransHis data.

**Table 1:** Bias risk in selected studies.

Type of bias	Case series studies						Case-control studies			Cohort studies			
	Koyi et al. 2002	Corner et al. 2005	Barros et al. 2006	Cajoto et al. 2009	Shresthra et al. 2010	Gonzalez- Baracala et al. 2014	Kubik et al. 2002	Hamilton et al. 2005	Iyen-Omofoman et al. 2013	Hoppe et al. 1977	Jones et al. 2007	Hippisley-Cox et al. 2011	Walter et al. 2015
Random sequence generation (selection bias)	•	•	•	•	•	•	•	•	•	•	•	•	•
Allocation concealment (selection bias)	•	•	•	•	•	•	•	•	•	•	•	•	•
Blinding of participants and personnel (performance bias)	ₒ	ₒ	ₒ	ₒ	ₒ	ₒ	ₒ	ₒ	ₒ	ₒ	ₒ	ₒ	ₒ
Blinding outcome assessment (detection bias)	ₒ	ₒ	ₒ	ₒ	ₒ	ₒ	ₒ	ₒ	ₒ	ₒ	ₒ	ₒ	ₒ
Incomplete outcome data (attrition bias)	ₒ	ₒ	ₒ	ₒ	ₒ	ₒ	ₒ	ₒ	ₒ	ₒ	ₒ	ₒ	ₒ
Selective reporting (attrition bias)	ₒ	ₒ	ₒ	ₒ	ₒ	ₒ	ₒ	ₒ	ₒ	•	•	•	ₒ
Other bias	ₒ	ₒ	ₒ	ₒ	ₒ	ₒ	ₒ	ₒ	ₒ	ₒ	ₒ	ₒ	ₒ

• Indicates high risk of bias

• Indicates uncertain risk of bias

ₒ Indicates low risk of bias

**Table 2:** Summary of selected studies.

Study (year)	Geographic area	Study design	Data source period	Sample demography and use of controls	Period of initial presentation	Characterisation of symptom	Staging or surgical management	Outcome measure
Koyi *et al*. 2002	Gaevleborg, Sweden	Prospective case series study using patient questio -nnaires completed within a specialist lung clinic	Patient questionnaire Jan 1997 –Dec 1999	364 participants–no controls	Not stated	Not characterised	Yes	Percentages
Corner *et al*. 2005	England, United Kingdom	Retrospective case series study interview triangulated with medical records	Medical Records (< 2 years before diagnosis)	22 participants (Male 54.5% Female 45.5%)–no controls	6–24 months prior to diagnosis	Not characterised	Yes, operability	Percentages
Barros *et al*. 2006	Curitian, Brazil	Retrospective case series study	Medical records Jan 1991- Dec 1997	268 participants–no controls	Not stated	Not characterised	Yes	Percentages
Cajoto *et al*. 2009 (SPANISH)	Santiago de composteka, Spain	Retrospective case series study	Medical records (codes) Jan 1997-Dec 1999	481 participants–no controls	Not stated	Not characterised	None	Percentages
Shrethra *et al*. 2010	Kathmandu Nepal	Retrospective case series study	Medical records July 2004—July 2008	174 participants–no controls	117.3 days prior to diagnosis	Not characterised	None	Percentages
Gonzalez- Barcala *et al*. 2014	Ponteveda Health Area, Spain	Retrospective case series study	Hospital records 1 June 2005–31 May 2008	358 patients–no controls	Unknown	Not characterised	Yes	Percentages
Kubik *et al*. 2002	Czech Republic	Case-control study	Patient interview questionnaire (not validated) April 1998—October 2000	All female 268 cases and 1076 control participants (not diagnosed with lung cancer), aged 25–89.	< 2 years	Yes, duration of presentation- looked at two presentations. Also, one associated feature, cough +/- phlegm.	None	Odds Ratio (adjusted for age, residence and education)
Hamilton *et al*. 2005	Exeter, United Kingdom	Case-control study controls	GP Medical records (codes) 1998–2002	247 cases and 1235 control participants no lung cancer with same presentation (GP/age/sex matched, age >40 years)	180 days to 2 years	Yes, associated symptoms as first and second symptom prior to diagnosis for seven specific symptoms.	None	Positive Predictive Value and Likelihood Ratios
Iyen-Omoforman *et al*. 2013	United Kingdom	Case-control study–controls from same general practice	GP Medical records (The Health Improvement Network database) Jan 2000—July 2009	12, 074 cases and 120,731 control participants	4–12 months 13–24 months	Yes, onset (period prior to diagnose) five specific symptoms	None	Odds Ratio, sensitivity, specificity
Hoppe *et al*. 1977 (GERMAN)	Hamburg, Germany	Retrospective Cohort study	Hospital records 1967–1974	20,000 participants in cohort	Not stated	Yes, duration of symptom prior to diagnosis	None	Percentages
Jones *et al*. 2007	United Kingdom	Retrospective Cohort study	Medical records (CPRD) Jan 1994—Dec 2000	4812 participants (>15 years) in cohort	6 months– 3 years	Assessed haemoptysis only as a lung cancer symptom.	None	Positive Predictive Value, Likelihood Ratios
Hippisley-cox *et al*. 2011	England and Wales, United Kingdom	Prospective Cohort study	GP Medical records (QResearch EMIS) Jan 2000- Sept 2010	3785 participants in cohort	< 2 years	Not characterised	None	Positive predictive value
Walter *et al*. 2015	England United Kingdom	Prospective Cohort study	Medical records and Questionnaire completed by interviewer Dec 2010 and Dec 2012	963 participants in cohort	28 days– 2 years	Yes, duration and presence of synchronous symptoms	None	Hazard ratios (adjusted for waiting time) and percentages

**Table 3:** Likelihood ratios for each presentation where indicted in selected studies.

Study (year)	Outcome measure	Symptom
Hamilton *et al*. 2005	LR+ (from raw data obtained from a referenced author)	Haemoptysis LR+13.2 (7.9–22) LR- 0.8 (0.76–0.86); Loss of weight LR+ 6.2 (4.5–8.6) LR- 0.76 (0.71–0.82); Loss of appetite LR+4.8 (3.3–7.0) LR- 0.84 (0.79–0.9); Dyspnoea LR+3.6 (3.1–4.3) LR- 0.52 (0.45–0.60); Chest or rib pain LR+3.3 (2.7–4.1) LR- 0.68 (0.61–0.75); Fatigue LR+2.3 (1. 9–2.9) LR-0.76 (0.7–0.84).
Iyen-Omoforman *et al*. 2013	LR+ calculated from published sensitivity and specificity	Haemoptysis 13.9; Cough 2.5; Chest/shoulder pain 1.9; Dyspnoea 5.4; Weight loss 3.6; Voice hoarseness 1.9.
Jones *et al*. 2007	LR+ and PPV	PPV 5.8% (5.0%-6.7%) and LR+ 116.7 (99.1–134.3) in men, and PPV 3.3% (2.6%-4.3%) and LR+ 153.1 (115.3–190.8) in women
Study (year)	Outcome measure where LR not available	Symptom
Kubik *et al*. 2000	OR (adjusted for age, residence, education and pack years)	Chronic cough 2.93 (2.03–4.22); Chronic phlegm 2.44 (1.59–3.76); Chronic phlegm < 2 years 4.74 (2.56–8.76); Chronic phlegm ≥2 years 1.43 (0.80–2.54); Dyspnoea 1.66 (1.18–2.34); Attacks of dyspnoea 1.10 (0.60–2.04).
Hippisley-cox *et al*. 2011	PPV	Current haemoptysis female 23.9 (20.6–27.6) male 21.5 (19.3–23.9); Current appetite loss female 4.14 (3.15–5.45) male 4.71 (3.69–6.00); Current weight loss female 4.52 (3.80–5.38) male 6.09 (5.33–6.95); New onset cough in last 12 months female 1.90 (1.56–2.32) male 1.47 (1.23–1.75)
Walter *et al*. 2015	HR (adjusted for waiting time paradox)	Coughing up blood (not included as less than 10 cases); Cough or worsening cough 43 weeks 1.16 (0.78–1.74) P = 0.46; Breathlessness or worsening 43 weeks 0.70 (0.45–1.08) P = 0.1; Chest/shoulder pain 43 weeks 1.79 (1.08–2.99) P = 0.03; Hoarseness 43 weeks 0.98 (0.48–2.01) P = 0.97; Decreased appetite 1.41 (0.78–2.53) P = 0.25; Unexplained weight loss 0.86 (0.43–1.71) P = 0.66; Fatigue or tiredness ‘unusual for you’ 1.16 (0.75–1.79) P = 0.49; Different ‘in yourself’ 1.52 (0.93–2.46) P = 0.09.

LR+ = positive likelihood ratio, PPV = positive predictive value, OR = odds ratio, HR = hazard ratio

#### Cohort studies

Retrospective cohort studies typically use data collected in the electronic health record: they usually exclude data collected in the 6–12 months before diagnosis to address the potential bias from including post-diagnosis symptoms and to minimise the influence of GPs preferentially coding possible lung cancer symptoms when considering this as a potential diagnosis. Cohort studies accounted for 31% of the selected studies. Jones and colleagues (2007), used a symptom-based approach to investigate all diagnoses associated with haemoptysis in a large general practice database (Clinical Practice Research Datalink) of 762,325 UK patients. Of the 4,812 new episodes of haemoptysis, 6.3% were subsequently diagnosed with lung cancer. This study also reported PPVs and positive likelihood ratios (LR+) as shown in Table 3 [13]. Hippisley-Cox and colleagues (2011) determined the hazard ratios for lung cancer in a risk assessment model that considered three clinical predictors (haemoptysis, loss of appetite and weight loss) presenting within 12 months prior to a lung cancer diagnosis. Risk of lung cancer was greatest in patients with haemoptysis: hazard ratio 23.9 (20.6–27.6) in females and 21.5 (19.3–23.9) in males, after adjustment for late-stage diagnosis and the associated shorter time-to-diagnosis, waiting time paradox [14]. Walter and colleagues (2015) used a prospective cohort study design and interviewed patients who had been referred to a specialist respiratory clinic by their GP. Half of the referred patients (49.3%) reported that they had presented to their GP with a single first symptom. Almost 40% (>37.8%) presented with more than one presenting symptom that worsened over time. Haemoptysis had the greatest causative association to lung cancer with an adjusted hazard ratio of 2.17 (1.63–2.89) (P = 0.00) [15].

#### Case-control studies

Case-control studies accounted for 23% of the selected studies and are limited in that the outcome measures such as PPVs cannot be generalised beyond the study. They are a product of the selection of cases and controls, not reflecting any natural population, although LRs may be valid for use with prior risk data. Kubik and colleagues (2000) assessed the diagnostic value of dyspnoea, chronic non-productive and productive cough. When adjusted for age, residence, education, and smoking pack-years, non-productive cough had an odds ratio of 2.93 (2.03–4.22), higher than that for productive cough 2.44 (1.59–3.76) and dyspnoea 1.66 (1.18–2.34) [16]. Hamilton and colleagues (2005) used a case-control study design to investigate the clinical features of lung cancer before diagnosis. Cases were identified retrospectively from local general practices and cancer registry databases. Symptoms reported within 180 days to 2 years prior to lung cancer diagnosis were assessed, to avoid bias, and compared with age and sex-matched control groups from the same general practices who did not have lung cancer. Seven specific presentations were assessed with the greatest positive likelihood ratios observed with haemoptysis 13.2 (7.9–22.0), then loss of weight 6.2 (4.5–8.6), loss of appetite 4.8 (3.3–7.0), dyspnoea 3.6 (3.1–4.3) and chest pain 3.3 (2.7–4.1)[17]. Iyen-Omofoman and colleagues (2013) used a routine data source (The Health Improvement Network). Clinical predictors were recorded during two time periods: 4–12 and 13–24 months prior to diagnosis. The highest odds ratio: 20.15 (16.24–25.01) was for haemoptysis presenting 4–12 months before diagnosis[18].

#### Case series studies

Case series studies accounted for 46% of the selected studies, the most common study design observed in this review but the least informative in relation to diagnostic value. The diagnostic value of these symptoms cannot be assessed because patients without lung cancer were not included in the study.

#### TransHis data

TransHis is an EHR specifically designed to capture the initial consultation as ‘Reason for Encounter’ (RfE) and maintain the episode of care structure as an ongoing prospective cohort study. The TransHis data were used to determine the relationship between lung cancer diagnosis and RfE, expressed as odds ratios. Cough followed by haemoptysis, dyspnoea, weight loss, chest pain and voice symptoms were the most prevalent RfEs in patients subsequently diagnosed with lung cancer (S5 Table). Constitutional symptoms (tiredness, weight loss, anorexia, fever and sweating) were collectively the third most common. As TransHis data is captured from routine care using a primary-care specific classification (ICPC2) and the odds ratios are relative to ‘all consulting patients’, we compared the outcomes with our selected studies.

When considering all the selected studies, haemoptysis, cough, dyspnoea, chest pain and constitutional symptoms were found to be the most prevalent presentations. In all studies haemoptysis, dyspnoea and cough were consistently the most predictive symptom for lung cancer.

#### Statistical analysis for diagnostic accuracy of clinical presentations associated with lung cancer

**Fig 2:**
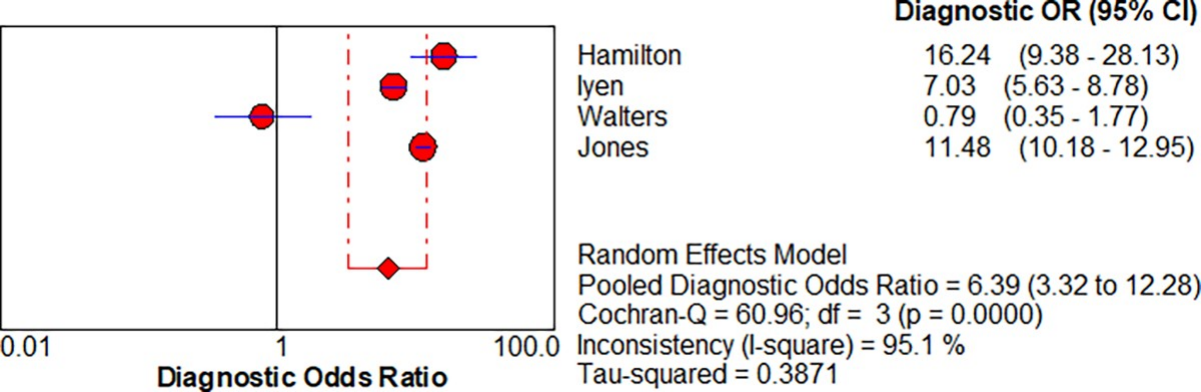
Forest plots with pooled diagnostic odds ratio (95% confidence interval) and weights calculated using a random effects model for haemoptysis in the diagnosis of lung cancer.

**Fig 3:**
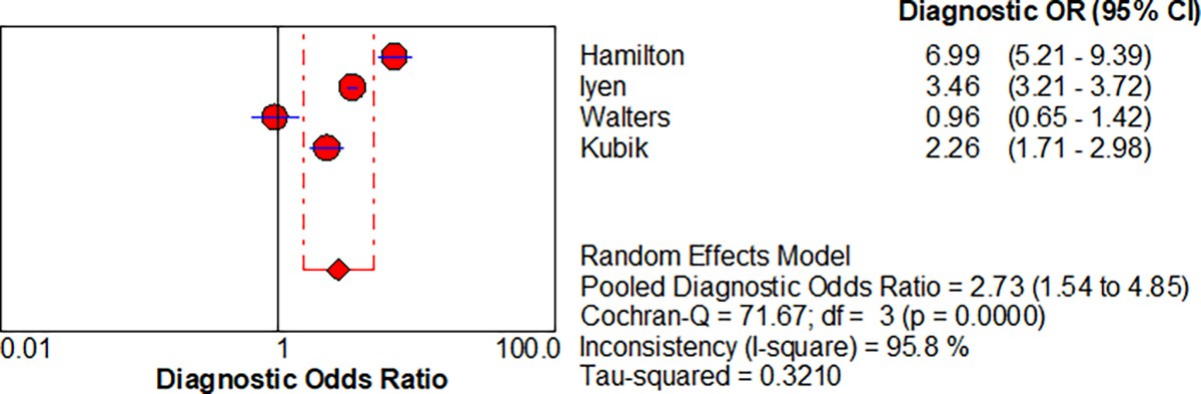
Forest plots with pooled diagnostic odds ratio (95% confidence interval) and weights calculated using a random effects model for Dyspnoea in the diagnosis of lung cancer.

**Fig 4:**
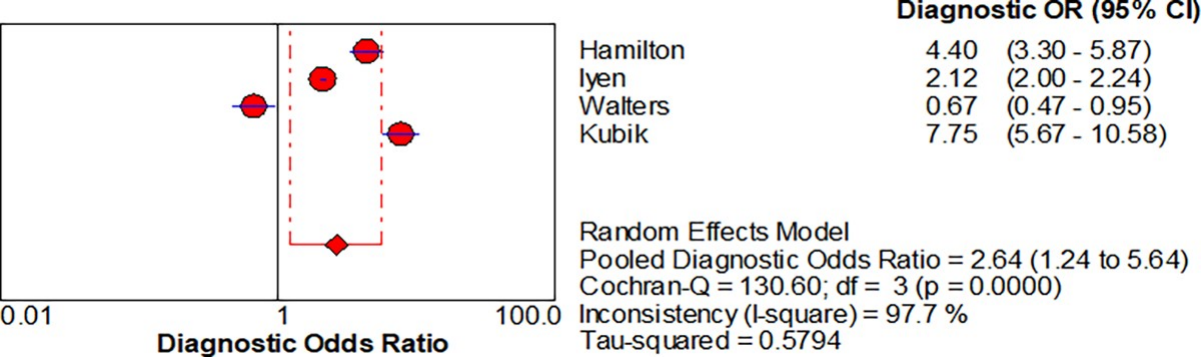
Forest plots with pooled diagnostic odds ratio (95% confidence interval) and weights calculated using a random effects model for Cough in the diagnosis of lung cancer.

**Fig 5:**
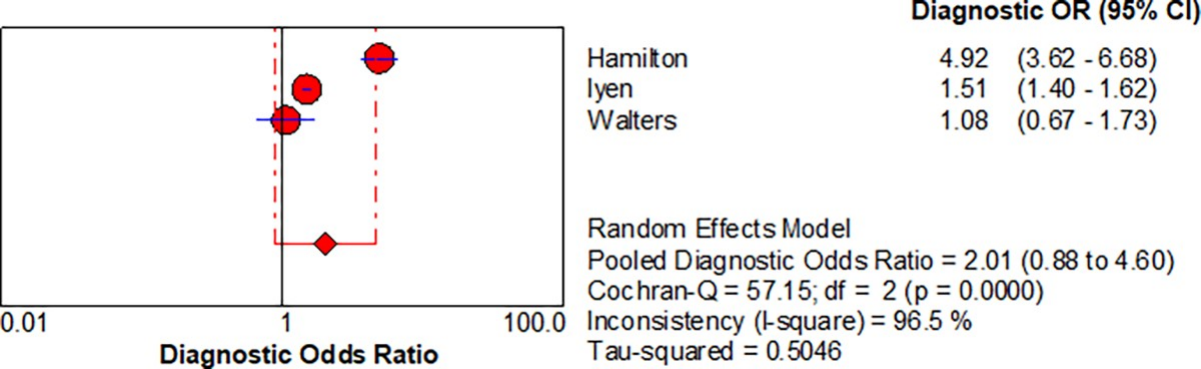
Forest plots with pooled diagnostic odds ratio (95% confidence interval) and weights calculated using a random effects model for Chest Pain in the diagnosis of lung cancer.

Five studies enabled us to assess the diagnostic accuracy of symptoms associated with lung cancer [16–18]. The pooled diagnostic odds ratios (DOR) for.haemoptysis, dyspnoea, cough and chest pain were 6.39 (3.32–12.28), 2.73 (1.54–4.85), cough 2.64 (1.24–5.64) and chest pain 2.01 (0.88–4.6) respectively, shown in Figs 2–5 respectively.

**Fig 6:**
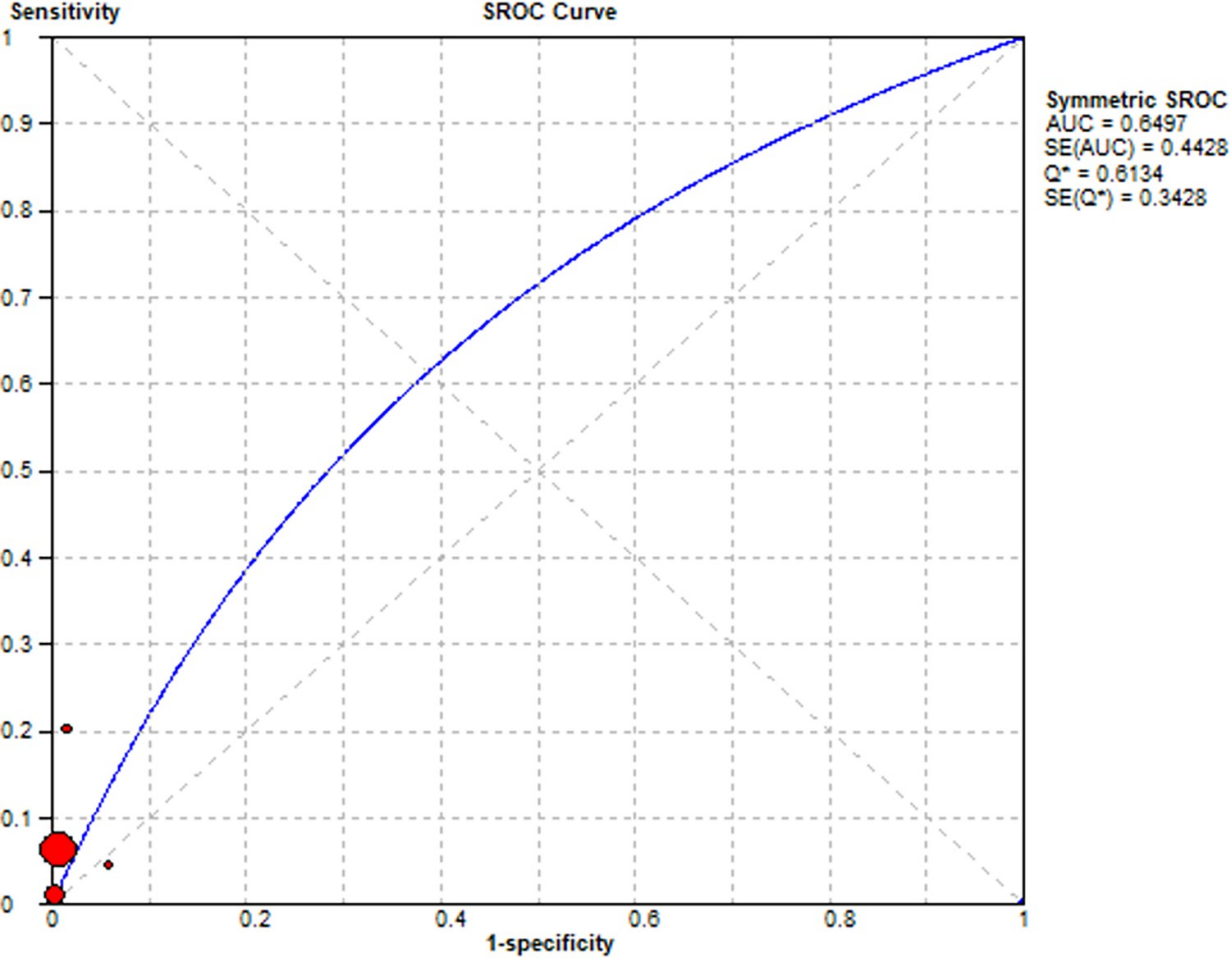
Summary Receiver Operator Curve for Haemoptysis as a diagnostic symptom in lung cancer.

**Fig 7:**
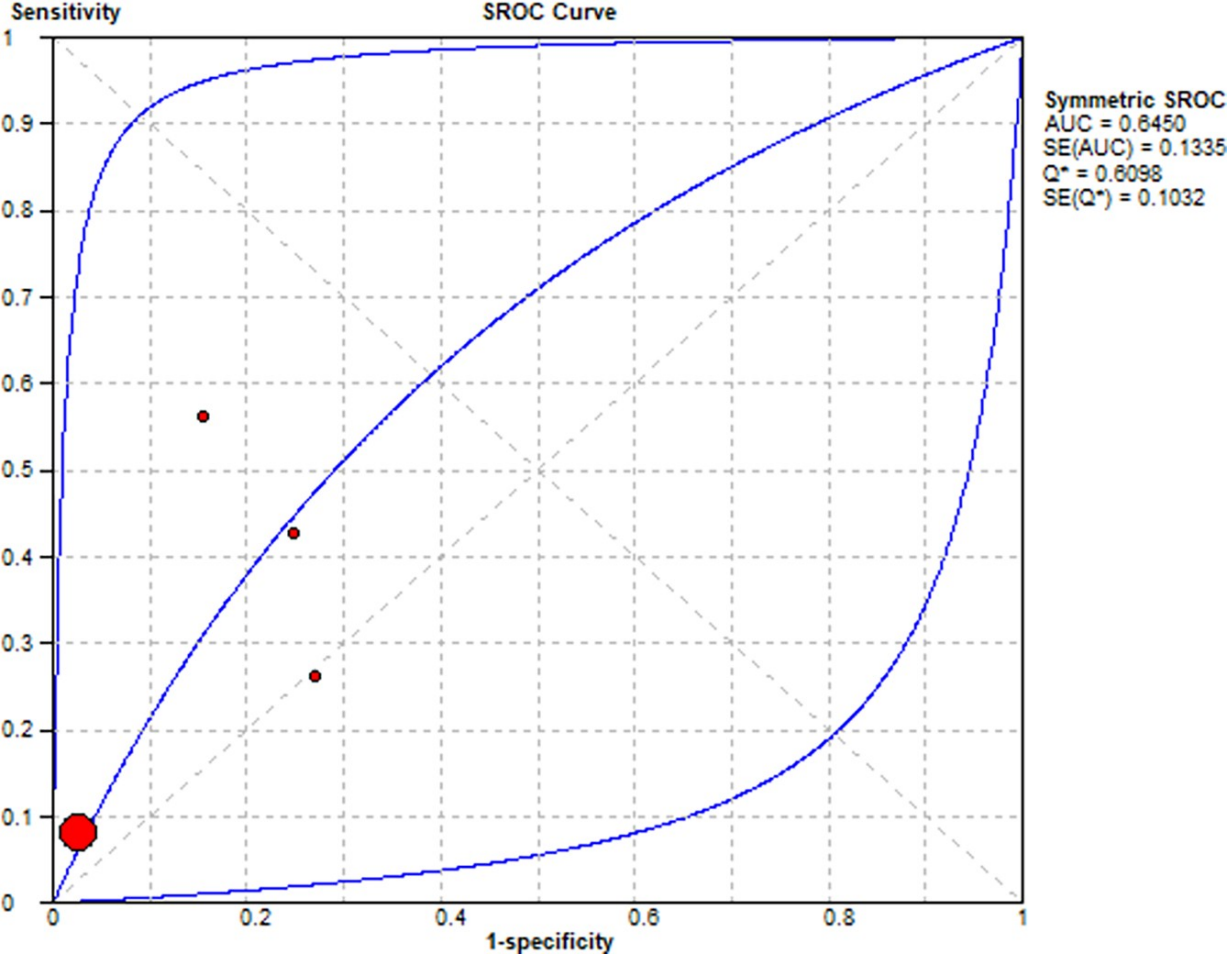
Summary Receiver Operator Curve for Dyspnoea as a diagnostic symptom in lung cancer.

**Fig 8:**
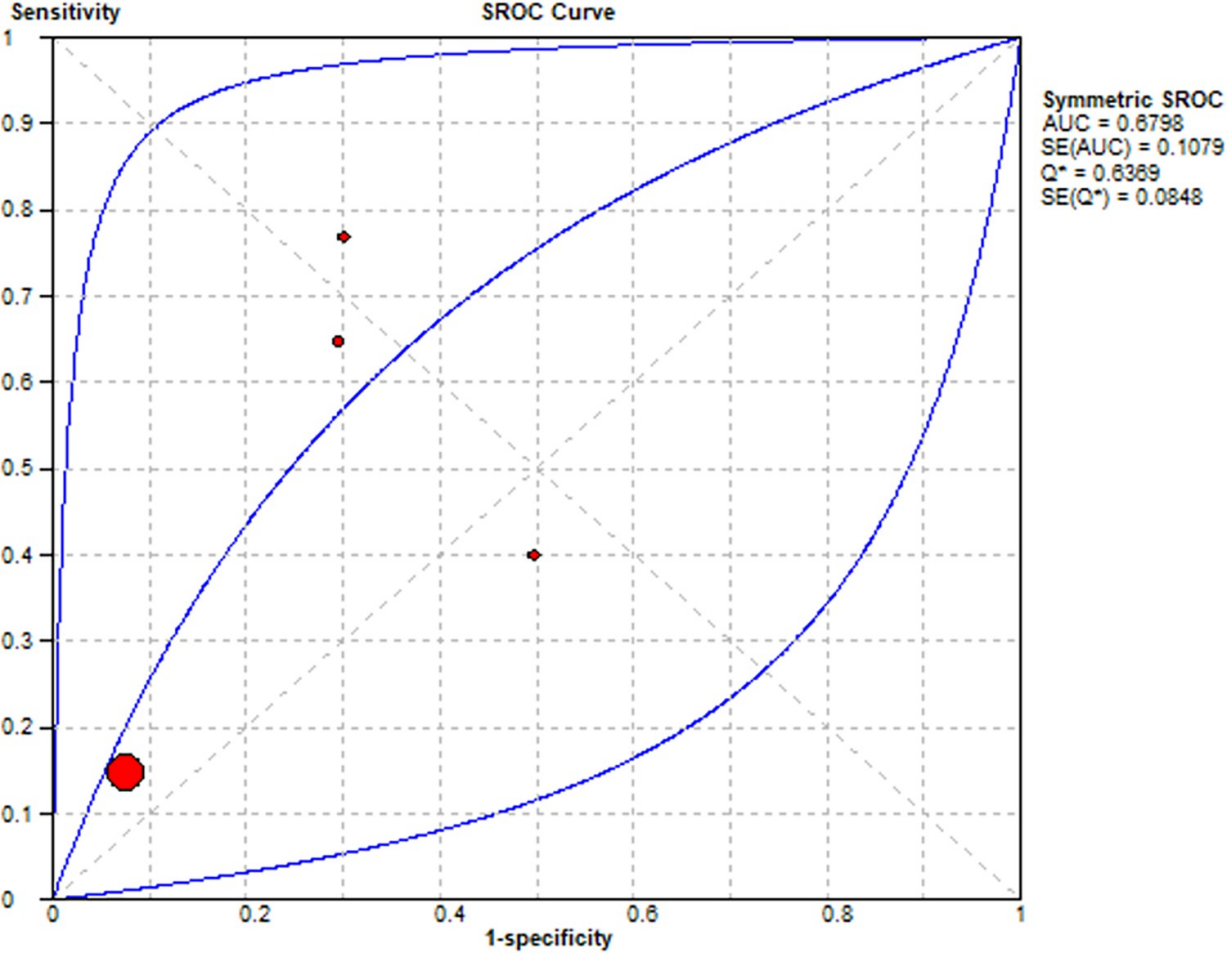
Summary Receiver Operator Curve for Cough as a diagnostic symptom in lung cancer.

**Fig 9:**
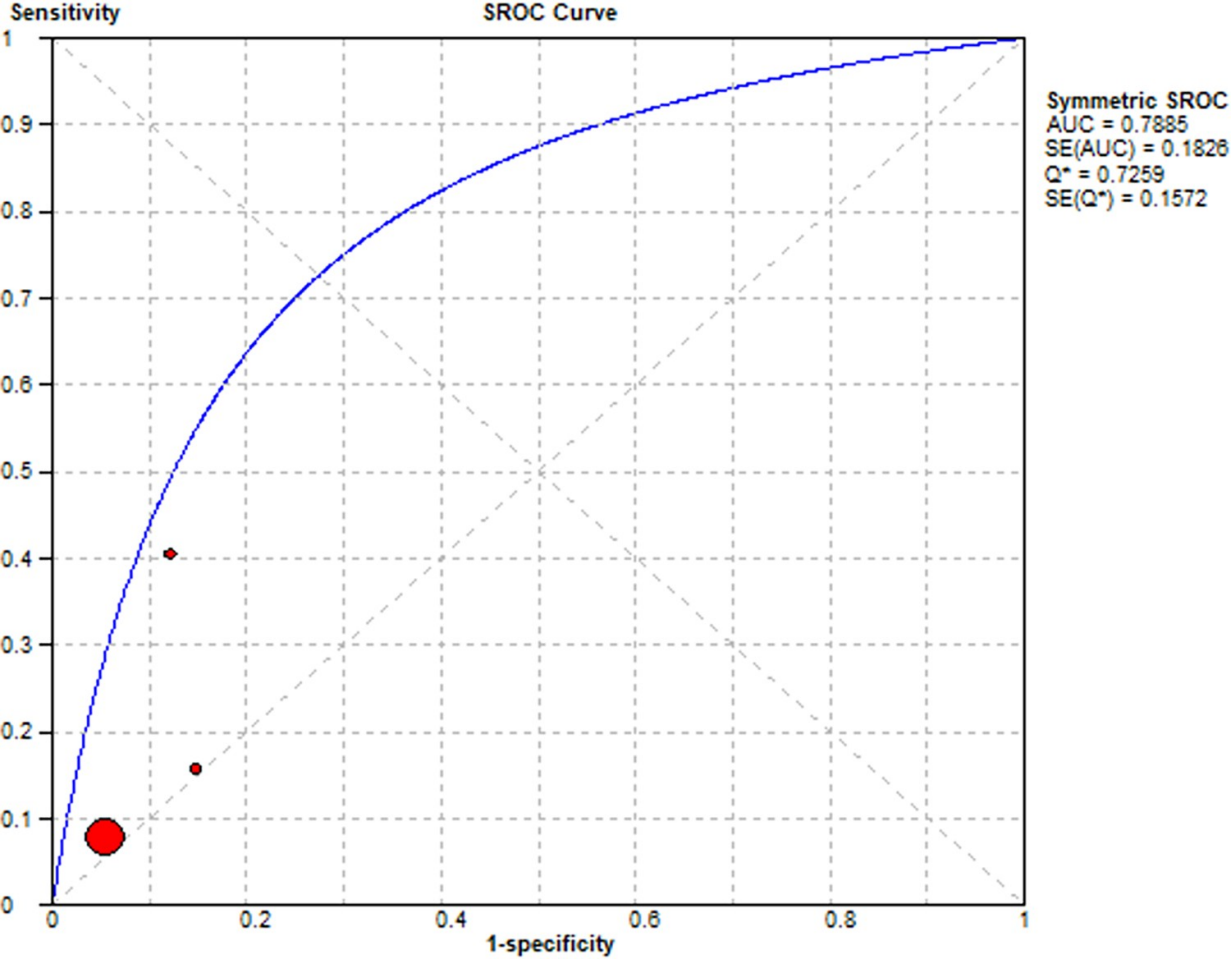
Summary Receiver Operator Curve for Chest pain as a diagnostic symptom in lung cancer.

Summary Receiver Operating Characteristic (SROC) curves were used to determine the predictive accuracy of each presentation in the diagnosis of lung cancer, using the area under the curve (AUC). Accuracy for lung cancer diagnosis was confirmed for haemoptysis AUC = 0.65, dyspnoea AUC = 0.65, cough AUC = 0.68 and chest pain AUC = 0.79. The SROC curves are summarised Figs 6–9 respectively.

The limited availability of studies that fit the criteria for diagnostic value, differences in study design and the differing thresholds for recording presence/absence of a symptom, shown in Tables 2 and 3, created the heterogeneity (I^2^) observed in the SROC curves Fig 3. We compared the overall diagnostic value of each presentation from the selected studies with measurable outcome data and TransHis data using likelihood ratios as shown in Table 4.

The symptom most likely to be observed in lung cancer vs non lung cancer patients is haemoptysis, followed by dyspnoea, cough and finally chest pain.

**Table 4:** Positive Likelihood Ratios (LR) for symptoms in Lung Cancer patients prior to diagnosis.

Symptom	Pooled positive likelihood ratio for selected studies (95% confidence intervals)	TransHis positive likelihood ratios (95% confidence intervals)
Haemoptysis	5.968 (3.183–11.189)	51.76 (24.91–107.56)
Dyspnoea	2.138 (1.350–3.385)	3.02 (1.72–5.32)
Cough	1.748 (1.290–2.369)	1.09 (0.69–1.73)
Chest pain	1.756 (0.953–3.237)	0.69 (0.17–2.73)

#### Staging at diagnosis of lung cancer

The tumour stage at diagnosis, or its operability, was indicated in only four of the thirteen studies and most of the diagnosed cases were inoperable or at stages IIa and above. Hence, 31% of selected studies described the prognostic benefits of symptom-based early diagnosis by including data on disease stage and operability at diagnosis [14, 15, 17–19].

In the most common form of lung cancer, non-small cell, the weighted means as a percentage of all cases in each study was calculated as follows: Stage I 10.7%, Stage II 6.9%, Stage III 43.2% and Stage IV 39.2%. These studies found that less than 8.2% of the lung cancer patients were amenable to surgery at diagnosis [20].

## Discussion

### Summary of findings

We found haemoptysis, had the greatest diagnostic value in both the selected studies and the TransHis database, followed by dyspnoea, cough and chest pain. The review also indicated that most of cancer patients are diagnosed at a late stage when there are limited surgical management options and less favourable clinical outcomes. More precise coding for symptoms and characterisation of symptoms, such as severity, timing and associated features, in electronic health records such as TransHis may provide sufficient evidence for early symptom-based diagnosis of lung cancer. It is hoped that the introduction of a new and global clinical vocabulary for electronic health records, SNOMED CT (Systematized Nomenclature of Medicine–Clinical Terms), will also contribute to better utilisation of electronic health records to improve evidence-based research. Although, codes will need to be carefully restricted to a classification of symptoms to enable calculation of odds ratios.

### Findings within the context of the current literature

To date, this is the only review to include a meta-analysis of clinical symptoms for the diagnosis of lung cancer. A previously published systematic review based on primary care data showed haemoptysis to be a predictor of lung cancer, but there were insufficient data to perform a meta-analysis [21]. We included studies where the index cases were identified in both primary care and secondary care studies as long as patients were referred by their GP. We made this decision on the basis that referral to a clinic for investigation of respiratory symptoms represents a cohort of people in whom the GP is considering cancer, and in the absence of better data on the evolution of symptoms over time, may yield useful LRs (but not PPVs). Our findings are consistent with previous findings that haemoptysis is predictive of lung cancer, but in addition demonstrates the diagnostic value of dyspnoea, cough and chest pain [15, 18, 21, 22].

Previously published studies suggest that efforts to expedite the diagnosis of symptomatic cancer are likely to benefit patients in terms of improved survival, earlier-stage diagnosis and improved quality of life [19, 23–27]. This review clearly identifies a place for symptom-based diagnosis, as the epidemiology of cancer symptoms is becoming better understood. Risk models that assess prior risk factors and then presenting symptoms could identify high-risk patients for early diagnosis [28].

### Strengths and weaknesses of the review

All selected studies used routine data sources, a cost-effective and powerful resource for evidence-based research. Though variability in the study designs creates heterogeneity, there was sufficient data to perform a meta-analysis and determine the diagnostic accuracy of clinical presentations associated with lung cancer. Five of the thirteen studies assessed the association of lung cancer with a specific set of symptoms and did not investigate all symptoms reported in lung cancer patients [13, 14, 16–18]. As a result, their findings may have missed other symptoms not already known to be associated with lung cancer. Each study provided demographic data on age, sex and smoking status; male smokers over 40 years were found to have the greatest incidence of lung cancer. However, routine data sources can also be subject to bias, such as missing data, coding inconsistencies, and work-up bias [29, 30] Thus, these studies can miss the complexities of the clinical assessment necessary for cancer diagnosis, for example weight loss was found to be the fifth most prevalent presentation prior to diagnosis and, in one study, it was observed even in operable disease, indicative of a presentation associated with early diagnosis [31]. In 62% of the selected studies, weight loss was grouped with constitutional symptoms, therefore, specific analysis of weight loss as an isolated symptom was not possible. More data are required for diagnostic assessment of weight loss because it may prove to be a cost-effective predictor of high-risk patients. These patients could be identified for further investigations to facilitate early cancer diagnosis.

### Implications for clinical practice and research

Case series studies represent a majority of the studies into symptoms associated with lung cancer, but this design has no diagnostic benefit because there are no controls. This highlights the importance of devising a study design that will produce clinically significant outcomes that will be of patient benefit.

Understanding the precise diagnostic value of symptoms is a powerful tool in clinical decision making [28]. Table 5 outlines three case scenarios where symptomology is considered in combination with prior risk [32] to establish indivualised risk and appropriate management. Up to 20% of all chest X-ray requests from primary care in patients subsequently diagnosed with lung cancer are negative [33, 34]. If we consider a high posterior risk of lung cancer, as shown in the Case C, even with a negative chest X-ray this patient still meets the criteria for urgent referral (PPV>3%), based on epidemiological risk factors and symptomology using Bayesian incorporation for posterior risk [17, 23, 35–39].

**Table 5:** Clinical case analysis using prior risk assessment and Bayesian incorporation of clinical symptoms to determine posterior risk.

Case	Sex	Age	Smoking status	Age started	Age stopped	Smoking duration	Smoking intensity (cigarettes/day)	Symptoms	LR+	Calculated prior risk %	PPV Calculated on the basis of individual posterior risk %	PPV based on presenting symptoms in the published cohort^[^^17^^]^*
A	M	68	Smoker	20	NA	48	10	cough + fatigue	3.45	1.86	6.14% Moderate risk	0.63% Low risk
Case A represents a low risk patient based on symptoms alone and therefore would not require further investigation or referral. When we take into account prior risk defined by age, sex, smoking status and intensity, this patient is at greater risk then the moderate risk patient in Case B below and does require further investigation (chest X-ray).												
B	F	61	Never	NA	NA	NA	NA	dyspnoea + haemoptysis	27.98	0.124	3.36% Moderate risk	4.90% Moderate risk
Case B represents a patient with moderate risk when considering symptoms alone. Here consideration of prior risk has little effect on the risk status.												
C	M	58	Ex	17	47	30	10	loss of appetite + haemoptysis	449.74	0.2697	54.88% High risk	45.28% High risk
Case C represents a high risk patient based on symptoms and even with a negative chest X-ray this patient would require further investigation to exclude lung cancer[17], as 20% of all chest X-ray requests from primary care in confirmed lung cancer patients are negative[33, 34]. The current cut-off of urgent cancer referrals in the UK is PPV>3% so this patient would be considered at high risk and should be investigated further, regardless of the chest X-ray findings.												

* from raw data provided by one of the referenced authors

Likelihood ratio = LR+ Positive predictive value = PPV

Over-reliance on chest X-ray findings and ignoring the patient’s prior risk could result in a missed diagnosis. This observation is reflected in the most recent NICE guidelines for referral of suspected cancer, it supports better primary care access to high-resolution imaging when indicated for high-risk patients [4].

Hamilton et al., 2005 investigated first and subsequent presenting symptom in lung cancer patients. Raw data from this study was utilised in the Bayesian model for risk of lung cancer described Table 5. Walters et al., 2015 looked at synchronous symptoms that occurred at the same time but did not define the specific symptom only the frequency of a single or synchronous symptom at first presentation.

In this systematic review we provide supporting evidence for four important symptoms for lung cancer diagnosis: haemoptysis, dyspnoea, cough and chest pain. It also highlights the difficulties with evaluating the diagnostic value of constitutional symptoms. For the diagnosis of relatively rare conditions such as cancer, population-based prospective cohort studies may never be feasible, hence, Walter and colleagues (2015) used selected high-risk patients. As we reach the limit of what we can be achieve with routine data in their current form, we must develop more defined and sophisticated criteria for clinical coding of symptoms and routine risk stratification of patients in real-time during clinical decision making [40, 41].

## Supporting information

S1 Table Prisma checklist. (DOCX)

S2 Table Quality assessment checklist. (DOCX)

S3 Table Database search outcomes. (DOCX)

S4 Table Summary of selected studies quality score, test description, bias, diagnostic sensitivity and specificity. (DOCX)

S5 Table Summary of real-time data from routine general practice for the most common presentions associated with lung cancer patients > 6 months before diagnosis (95% confidence intervals)–Netherlands, Malta, Serbia and Japan since 1995 and including 19700 patients. (DOCX)
