# Correction: Is symptom-based diagnosis of lung cancer possible? A systematic review and meta-analysis of symptomatic lung cancer prior to diagnosis for comparison with real-time data from routine general practice

**DOI:** 10.1371/journal.pone.0210108

**Published:** 2018-12-28

**Authors:** The PLOS ONE staff

As a result of the typesetting process, Figs 4–9 and Table 4 have been incorrectly placed in the Discussion section in the PDF version of the article. The publisher apologizes for the error.

In the PDF version of the article, Figs 4–5 should appear in numerical order directly after Fig 3 in the “Statistical analysis for diagnostic accuracy of clinical presentations associated with lung cancer.” sub-section of the Results section.

Figs 6–9 should appear in numerical order between the second and third paragraphs of the “Statistical analysis for diagnostic accuracy of clinical presentations associated with lung cancer.” sub-section of the Results section.

Table 4 should appear after the final paragraph of the “Statistical analysis for diagnostic accuracy of clinical presentations associated with lung cancer.” sub-section of the Results section.
