# Supplementary material for: Is symptom-based diagnosis of lung cancer possible? A systematic review and meta-analysis of symptomatic lung cancer prior to diagnosis for comparison with real-time data from routine general practice
Source: PLoS One. 2018 Nov 21;13(11):e0207686. doi: 10.1371/journal.pone.0207686 (PMC6248994; doi:10.1371/journal.pone.0207686)
Supplement: S2 Table — (DOCX) [file pone.0207686.s002.docx]

| **S2 Table, Quality assessment checklist** |
| --- |
| Study structure (sampling bias)   - Is the research questions or study aims and rational clearly stated? (1 mark) - Did the study avoid inappropriate participant exclusion? (1 mark) - Was recruitment based on presenting symptoms and/or a specific diagnosis? (1 mark) - What type of study is it, was data collection planned before the index test and reference standard were performed (prospective study)?( 4 marks) Or after (retrospective study)? (2 mark) |
| **Reference standard and rational (incorporation bias)**   - Describe who executed the index and reference tests? Did those executing the index tests and reference standard have the appropriate training and expertise? (1 mark) - Is there a definition and rationale for the units, cut-offs and/or categories of the results of the index tests and the reference standard? Also, if a threshold was used, was it pre-specified? (1 mark) - Were the readers of the index tests and reference standard blinded (masked) to the results of the other test. Describe any other clinical information available to the readers. Were the index test results interpreted without knowledge of the results of the reference standard? (1 mark) |
| **Study type, representative and applicable**   - When was the study performed, including beginning and end dates of recruitment? (1 mark) - What methods were used for calculating or comparing measures of diagnostic accuracy? Were there statistical methods used to quantify uncertainty (e.g. 95% confidence intervals). Is the reference standard likely to correctly classify the target condition? (1 mark) - What if any methods were used for calculating test reproducibility? (1 mark) - Where characteristics of the study population included (at least information on demography such as age, gender, spectrum of presenting symptoms)? (1 mark) - Were all patients included in the analysis? Did the number of participants satisfying the criteria for inclusion all undergo the index and the reference test? Describe why participants failed to undergo either test using a flow diagram. (1 mark) - How were indeterminate results, missing data and outliers of the index tests handled? (1 mark) |
| **Timing and data collation and analysis**   - Was there an appropriate interval between index test and reference test (standard)? (1 mark) - Did the study cross-tabulate results of the index test (including indeterminate and missing results) with the results of the reference standard test? (1 mark) |
| **Clinical application outlined and safety**   - Any adverse events from performing the index test or the reference standard test? (1 mark) |
