# Supplementary material for: Is symptom-based diagnosis of lung cancer possible? A systematic review and meta-analysis of symptomatic lung cancer prior to diagnosis for comparison with real-time data from routine general practice
Source: PLoS One. 2018 Nov 21;13(11):e0207686. doi: 10.1371/journal.pone.0207686 (PMC6248994; doi:10.1371/journal.pone.0207686)
Supplement: S3 Table — (DOCX) [file pone.0207686.s003.docx]

| **S3 Table. Database search outcomes** | | |
| --- | --- | --- |
| **Database and search date**  **24^th^ September 2017** | **Search terms** | **Results** |
| Ovid MEDLINE(R) 1946 to September Week 2 2017 | 1. exp Lung Neoplasms/di, ep, et [Diagnosis, Epidemiology, Etiology]  2. (sign or signs or symptom* or presentation).mp. [mp=title, abstract, original title, name of substance word, subject heading word, keyword heading word, protocol supplementary concept word, rare disease supplementary concept word, unique identifier]  3. (diagnos* or detection or differential).mp. [mp=title, abstract, original title, name of substance word, subject heading word, keyword heading word, protocol supplementary concept word, rare disease supplementary concept word, unique identifier]  4. 1 and 2 and 3  ===================================  NB Lung cancer in Medline =MeSH term lung neoplasm | 6142 |
| Embase1974 to 2017 Week 33 (September 2017) | 1. *lung cancer/di, ep [Diagnosis, Epidemiology]  2. (sign or signs or symptom* or presentation).mp. [mp=title, abstract, subject headings, heading word, drug trade name, original title, device manufacturer, drug manufacturer, device trade name, keyword]  3. (diagnos* or detection or differential).mp. [mp=title, abstract, subject headings, heading word, drug trade name, original title, device manufacturer, drug manufacturer, device trade name, keyword]  4. 1 and 2 and 3 | 3330 |
| HMIC Health Management Information Consortium 1979 to September 2017 | 1. exp Lung cancer/  2.(sign or signs or symptom* or presentation) mp. [mp=title, other title, abstract, heading words]  3. (diagnos* or detection or differential).mp. [mp=title, other title, abstract, heading words]  4. 1 and 2 and 3 | 70 |
| Web of science 1970 to September 2017 | TS=(lung cancer) AND TS=(signs OR sign OR symptoms* OR presentation) AND TS=(diagnos* OR detection OR differential) | 4611 |
| Cumulative Index to Nursing and Allied Health Literature 1990 to September 2017 | Database - CINAHL  Interface - EBSCOhost Research Databases  Search Screen - Advanced Search  Search modes - Boolean/Phrase  TX (lung cancer or "lung neoplasm") AND ( diagnos* or detection or differential ) AND ( sign or symptom or presentation) | 691 |
| British Nursing Index (1940- current, decades) | (lung cancer) OR (lung neoplasm) AND (sign OR signs OR presentation OR symptom*) AND (detection OR diagnos* OR differential) | 6194 |
| Cochrane library 1993 to September 2017 | #1 MESH [Lung Neoplasms] explode all trees and with qualifier(s): [Diagnosis - DI, Epidemiology - EP, Etiology - ET]  #2 lung cancer  #3 diagnos” or "detection" or "differential"  #4 sign or symptom or presentation  #5 #1 or #2 and #3 and #4 | 866 |
