# Supplementary material for: Is symptom-based diagnosis of lung cancer possible? A systematic review and meta-analysis of symptomatic lung cancer prior to diagnosis for comparison with real-time data from routine general practice
Source: PLoS One. 2018 Nov 21;13(11):e0207686. doi: 10.1371/journal.pone.0207686 (PMC6248994; doi:10.1371/journal.pone.0207686)
Supplement: S4 Table — (DOCX) [file pone.0207686.s004.docx]

| **S4 Table. summary of selected studies quality score, test description, bias, diagnostic sensitivity and specificity** | | | | | | | |
| --- | --- | --- | --- | --- | --- | --- | --- |
| **Study (year)** | **Quality score** | **Blinded study**  **(review bias)** | **Index test Positive**  **Details of clinical presentation** | **Reference standard** | **Sensitivity (95% CI)** | **Specificity (95% CI)** | **Bias** |
| **Koyi *et al.* 2002** | 11/20 | Unknown | Yes | Histology and cancer registry | No | No | Verification bias |
| **Corner *et al.* 2005** | 13/20 | No | Yes | Histology | No | No | Verification bias |
| **Barros *et al.***  **2006** | 9/20 | No | Yes | Histology | No | No | Verification bias |
| **Cajoto *et al.***  **2009**  **SPANISH** | 12/20 | No | Yes | Histology | No | No | Verification bias |
| **Shrethra *et al.* 2010** | 9/20 | No | Yes | Histology | No | No | Verification bias |
| **Gonzalez-**  **Barcala *et al*. 2014** | 12/20 | No | Yes | Histology | No | No | Verification bias |
| **Kubik *et al.* 2002** | 12/20 | No | Yes | Histology | Yes | Yes | Verification bias  Spectrum bias |
| **Hamilton *et al.* 2005** | 12/20 | Unknown | Yes | Histology | Yes | Yes | Verification bias  Spectrum bias |
| **Iyen-Omoforman *et al.* 2013** | 14/20 | Unknown | Yes | Histology | Yes | Yes | Verification bias  Spectrum bias |
| **Hoppe *et al.* 1977**  **GERMAN** | 13/20 | Unknown | Yes | Histology | No | No | Spectrum bias  Double gold bias |
| **Jones *et al.* 2007** | 11/20 | Unknown | Yes | GP records | Yes | Yes | Verification bias |
| **Hippisley-cox *et al.* 2011** | 18/20 | Unknown | Yes | GP records | Yes | Yes | Verification bias |
| **Walter *et al.* 2015** | 14/20 | Unknown | Yes | Histology and hospital notes | Yes | Yes | Verification bias  Spectrum bias  Double gold standard bias |
