# Supplementary material for: Is symptom-based diagnosis of lung cancer possible? A systematic review and meta-analysis of symptomatic lung cancer prior to diagnosis for comparison with real-time data from routine general practice
Source: PLoS One. 2018 Nov 21;13(11):e0207686. doi: 10.1371/journal.pone.0207686 (PMC6248994; doi:10.1371/journal.pone.0207686)
Supplement: S5 Table — (DOCX) [file pone.0207686.s005.docx]

| **S5 Table.** **Summary of real-time data from routine general practice for the most common presentations associated with lung cancer patients > 6 months before diagnosis** 5% confidence intervals) – Netherlands, Malta, Serbia and Japan since 1995 and including 19700 patients. | | | | | | | | | |
| --- | --- | --- | --- | --- | --- | --- | --- | --- | --- |
| Reason for encounter | No. per 1000 pts | Sensitivity | Specificity | LR+ | LR- | PV+ | PV- | Odds Ratio | |
| Cough | 16 | 0.13 | 0.88 | 1.09 | 0.99 | 0.00 | 0.99 | 1.11 | |
| Dyspnoea | 11 | 0.09 | 0.97 | 3.02 | 0.94 | 0.00 | 0.99 | 3.22 | |
| Haemoptysis | 7 | 0.06 | 1.00 | 51.7 | 0.9 | 0.03 | 0.99 | 54.8 | |
| Pain attributed to the respiratory system | 4 | 0.03 | 0.99 | 5.88 | 0.97 | 0.00 | 0.99 | 6.05 | |
| General weakness/tiredness | 4 | 0.03 | 0.97 | 1.13 | 1.00 | 0.00 | 0.99 | 1.14 | |
| Shoulder symptoms/complaints | 4 | 0.03 | 0.97 | 1.13 | 1.00 | 0.00 | 0.99 | 1.14 | |
| Symptom/complaint of the throat | 4 | 0.03 | 0.95 | 0.68 | 1.02 | 0.00 | 0.99 | 0.67 | |
| Voice symptoms /complaints | 3 | 0.02 | 0.99 | 4.62 | 0.98 | 0.00 | 0.99 | 4.71 | |
| Weight loss | 2 | - | - | - | - | - | - | - | |
| Chest symptom/complaints | 2 | - | - | - | - | - | - | - | |
| Anorexia/ eating problems | 1 | - | - | - | - | - | - | - | |
| Fever | 1 | - | - | - | - | - | - | - | |
| Sweating problem | 1 | - | - | - | - | - | - | - |  |
